# Steady-State Kinetic Modeling Constrains Cellular Resting States and Dynamic Behavior

**DOI:** 10.1371/journal.pcbi.1000298

**Published:** 2009-03-06

**Authors:** Jeremy E. Purvis, Ravi Radhakrishnan, Scott L. Diamond

**Affiliations:** 1 Institute for Medicine and Engineering, University of Pennsylvania, Philadelphia, Pennsylvania, United States of America; 2 Genomics and Computational Biology Program, University of Pennsylvania, Philadelphia, Pennsylvania, United States of America; 3 Department of Bioengineering, University of Pennsylvania, Philadelphia, Pennsylvania, United States of America; 4 Department of Chemical and Biomolecular Engineering, University of Pennsylvania, Philadelphia, Pennsylvania, United States of America; University of Illinois at Urbana-Champaign, United States of America

## Abstract

A defining characteristic of living cells is the ability to respond dynamically to external stimuli while maintaining homeostasis under resting conditions. Capturing both of these features in a single kinetic model is difficult because the model must be able to reproduce both behaviors using the same set of molecular components. Here, we show how combining small, well-defined steady-state networks provides an efficient means of constructing large-scale kinetic models that exhibit realistic resting and dynamic behaviors. By requiring each kinetic module to be homeostatic (at steady state under resting conditions), the method proceeds by (*i*) computing steady-state solutions to a system of ordinary differential equations for each module, (*ii*) applying principal component analysis to each set of solutions to capture the steady-state solution space of each module network, and (*iii*) combining optimal search directions from all modules to form a global steady-state space that is searched for accurate simulation of the time-dependent behavior of the whole system upon perturbation. Importantly, this stepwise approach retains the nonlinear rate expressions that govern each reaction in the system and enforces constraints on the range of allowable concentration states for the full-scale model. These constraints not only reduce the computational cost of fitting experimental time-series data but can also provide insight into limitations on system concentrations and architecture. To demonstrate application of the method, we show how small kinetic perturbations in a modular model of platelet P2Y_1_ signaling can cause widespread compensatory effects on cellular resting states.

## Author Summary

Cells respond to extracellular signals through a complex coordination of interacting molecular components. Computational models can serve as powerful tools for prediction and analysis of signaling systems, but constructing large models typically requires extensive experimental datasets and computation. To facilitate the construction of complex signaling models, we present a strategy in which the models are built in a stepwise fashion, beginning with small “resting” networks that are combined to form larger models with complex time-dependent behaviors. Interestingly, we found that only a minor fraction of potential model configurations were compatible with resting behavior in an example signaling system. These reduced sets of configurations were used to limit the search for more complicated solutions that also captured the dynamic behavior of the system. Using an example model constructed by this approach, we show how a cell's resting behavior adjusts to changes in the kinetic rate processes of the system. This strategy offers a general and biologically intuitive framework for building large-scale kinetic models of steady-state cellular systems and their dynamics.

## Introduction

Computational models help quantify the reaction dynamics and regulatory modes in complex biochemical systems [1]–[5], particularly when a system is so intricate that its behavior cannot be predicted by intuition alone. The building blocks for constructing large reaction networks are often available in numerous databases [6]–[9] and journal archives. Here, one can obtain many of the experimentally-derived elementary reaction steps, kinetic constants, or rate laws for individual steps in a given biochemical system or pathway. Despite this wealth of information, however, compiling these data to construct models with accurate system-wide behavior represents a significant challenge in systems biology [10],[11]. Comprehensive models of metabolism have been successfully developed for microbial systems [5],[12],[13] and certain eukaryotic cell types [14]–[16]. These constraint-based models [17] are often represented by stoichiometric networks that lack an explicit description of substrate concentrations, reaction mechanisms, or the transient behavior of the system. Although various strategies have been proposed to incorporate these features into large-scale models [18],[19], the task of assembling complex kinetic models with nonlinear dynamics remains a difficult problem. One of the major obstacles to building accurate kinetic models is the number of unknown parameters in the model that must be estimated using experimental datasets [19], which themselves are often massive, incomplete, noisy, and/or imperfect [20]. A number of parameter estimation methods, such as genetic programming, simulated annealing, and various gradient-based routines [21],[22], have been proposed to infer unknown quantities in biochemical models. Most of these methods address the problem of estimation in purely abstract terms and do not take into account the unique mathematical features of biochemical systems, such as a well-characterized kinetic subsystem (e.g., the dynamics properties of an ion channel [23]). Estimated parameters must still meet constraints imposed by the other experimentally measured parameters in the model.

To address these challenges, we propose a strategy for assembling large kinetic networks that retain the nonlinear dynamics governing individual reactions in the system. The key features of the method are: (*i*) restriction of steady-state values by subsystem kinetics, (*ii*) reduction of the steady-state solution space by principal component analysis (PCA), and (*iii*) combination of independently constructed submodels (modules). The first feature is a Monte Carlo sampling over unknown concentrations with fixed kinetic parameters derived from the literature. The opposite strategy has been used in microbial systems to restrict kinetic parameters based on species concentrations [12]. The second feature, reduction of the steady-state space by PCA, has been applied previously for metabolic systems described by a stoichiometry matrix [5],[13], but not, to our knowledge, for nonlinear systems. In the last step, a full model representation is assembled by combining PCA-reduced, steady-state solutions from each module to form a combined steady-state solution space for the entire system. This global space may then be searched for solutions with accurate time-dependent behavior using any number of established routines 22,24.

The method exploits three properties common to many biological systems: modularity, homeostasis, and known quantitative kinetic relationships among interacting molecular components. Interestingly, this physiology-inspired approach enforces natural constraints on the range of allowable system states and allows one to monitor shifts in steady states due to kinetic perturbations. To illustrate the method with an example, we show how 77 reactions from 17 primary data sources were integrated to construct an accurate model of intracellular calcium and phosphoinositide metabolism in the resting and activated human platelet. Finally, we extend our analysis of this modeling approach by examining the steady-state characteristics of a system that is affected by changes in kinetic rate constants.

## Materials and Methods

### Model Definitions and Requirements

**Figure 1:**
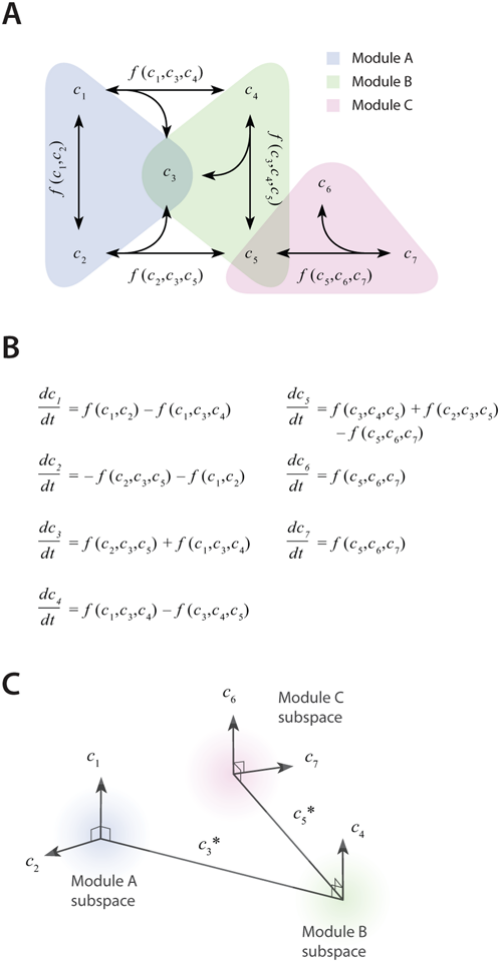
Example structure of model topology, ODEs, and concentration space. (A) The model topology defines the state transitions (arrows) and rate equations (*f*) that determine how molecules are interconverted. This example model is organized into three overlapping modules, with molecules 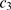 and 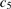 each occurring in two modules. Corresponding (B) ODEs and (C) concentration space for the example topology in panel A. Each of the 7 molecules occupies a separate linear dimension, with each module comprising a subspace of the full 7-dimensional space. Modules that share a common molecule have intersecting subspaces.

Our method builds upon a common representation of biochemical reaction networks [25] consisting of a system of ordinary differential equations (ODEs). In this paradigm, the concentration of each molecule in the system changes with time as a function of the instantaneous values of other concentrations and fixed kinetic parameters in the model. We separate this model description into two parts: The *concentration vector* (CV) of the model refers to the set of all molecule concentrations at a given instant in time and is denoted by the vector **c**:
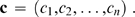
(1)The model *topology* refers to the entire set of kinetic parameters and rate equations that determine how these concentrations evolve with time. Mathematically, this is represented by the vector function ***f***, which defines the rate of change of **c** with time as a function of the model concentrations and rate parameters:
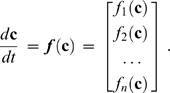
(2)The functional form of each 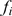 is a sum of rate equations for each reaction that consumes or produces 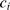 and will generally vary for each molecule. Typical functional forms for *f* may include, for example, a series of Michaelis-Menten or nonlinear rate expressions. A simple reaction topology is shown in Figure 1A with corresponding ODEs in Figure 1B. It is useful to separate a large model into two or more *modules* with subset CVs that overlap at reaction edges, as shown in Figure 1A.

Often, the topology of a biological system is better characterized than its CV [17]. For example, the major protein-protein interactions in a signaling pathway may be deduced from mutation or knock-out studies, providing a molecular wiring diagram that links together the various components in the network. For each of these interactions, purified enzymes may be used to measure the strength of the interaction *in vitro* or to measure the rate of some enzyme-catalyzed reaction in the system. An important caveat is that the kinetic rate constants within the cellular milieu (the cell context) may be different from those obtained in an *in vitro* experiment with purified components. In contrast, it is generally more difficult to accurately measure the absolute abundance of intracellular enzymes or metabolites *in vivo*, although progress is being made in this area [26]. Our method thus assumes that the topology of a given system is known and that the unknown set of concentrations exists in a linear space of dimension *n* in which each species 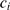 comprises a separate dimension (Figure 1C). The ultimate goal of the method is to efficiently search this *concentration space* to find a set of values that, when combined with the fixed topology, renders the full model consistent with known resting states and experimental time-series data obtained by perturbation of the cell.

A special situation arises when 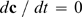 in equation (2). Under these conditions, the model is said to be at steady state, and the vector 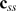 is a steady-state solution to the system of ODEs. If ***f*** contains nonlinear terms, there may be an infinite number of steady-state solutions for the system of ODEs [25]. This set of solutions occupies some nonlinear subspace of the concentration space exemplified in Figure 1C. To guarantee that nonzero steady-state solutions may be found, the method requires the model topology (and all module topologies) to be balanced, meaning that the production and consumption of each molecule must be equal so that the total mass of the system is conserved. This steady-state assumption [17] is a common constraint in stoichiometric modeling and metabolic flux analysis and is conceptually related to the biological phenomenon of homeostasis [27], in which opposing processes are coordinated to maintain the stability of a cell or organism. For example, a nerve cell may maintain a constant electrochemical gradient by continually transporting ions across a lipid membrane.

## Results/Discussion

### Reduction of Modular Kinetic Networks

**Figure 2:**
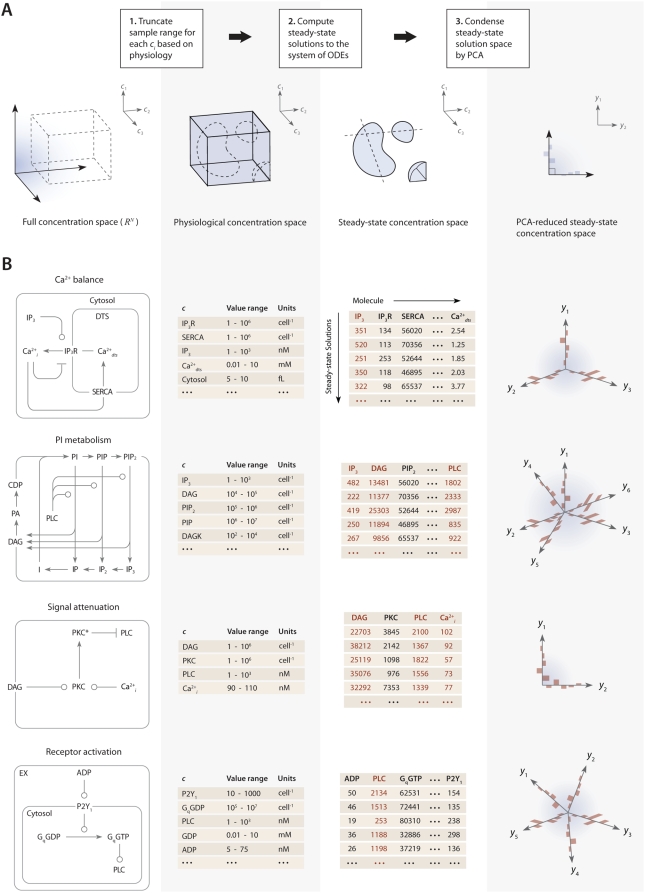
Steps in dimensionality reduction of steady-state modules and example from platelet signaling model. (A) Steps in dimensionality reduction of kinetic modules: (1) Restrict value ranges for each *c* to physiologically realistic ranges. (2) Compute multiple steady-state solutions to the model ODEs using initial guesses sampled randomly from the defined distribution. (3) Reduce the dimensionality of the steady-state solution set by PCA. (B) Results obtained from modular reduction 4 kinetic modules in a platelet signaling model. For each module, a fixed topology was combined with initial guesses from the defined distribution and simulated until equilibrium was reached (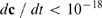) using 10^9^ initial guesses for 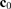. Specific concentrations within these steady-state solutions were compared to experimentally measured values, and solutions with low error (±10% of known concentration values) for these elements were selected as “points” in the steady-state concentration space. PCA was then applied to transform these points to a new coordinate set that maximally covers the space of steady-state solutions.

The first phase of the method involves generating a compact representation of the steady-state solutions for each module. The steps for module reduction are outlined in Figure 2A. First, conservative bounds are chosen for **c** based on physiological and practical considerations. For example, a regulatory enzyme is expected be present in at least one copy per cell and not to exceed an intracellular concentration of one molar. Knowledge about the physical size of the system is useful in this step to convert a raw copy number to a concentration. For small systems, this information can provide a rigid lower bound on unknown concentrations [28]. For example, a single molecule in a 6 fL platelet has a concentration of 4 nM. Also, because molecular concentrations can span several orders of magnitude, it is often more efficient to delineate this range of values on a logarithmic scale rather than a linear scale.

Once the sampling distribution for **c** has been defined, steady-state solutions (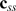) for each module are calculated using fixed kinetic parameters for each reaction in the module obtained from the literature [6],[8],[9], novel kinetic experiments, or estimation. For this step, each initial guess 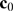 is sampled from the distribution for **c** and combined with the predetermined topology. The combination of fixed rate equations, fixed parameters, and 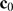 forms a well-posed initial value problem,
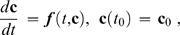
(3)that may be computed using a numerical solver [29]. For non-oscillating systems, steady-state solutions may be obtained by simulating the system until equilibrium is reached (i.e., until 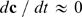 ). Alternatively, one may use any number of multidimensional root-finding routines, such as those available in the GNU Scientific Library [30], to find the closest *n*-dimensional root to the vector function ***f*** using starting guess 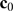.

In the third step, a large collection of steady-state solutions for each module is subjected to principal component analysis (PCA). A sample size of 1000 points per unknown concentration is generally sufficient to minimize error due to over-fitting [31]. PCA is then used to transform these points to a new coordinate set that optimally covers the space of steady-state solutions using the fewest number of dimensions. For example, if two molecule concentrations in the steady-state space are highly correlated due to participation in the same reaction, PCA will locate a single dimension to represent each pair of points in the transformed space. Ultimately, these new dimensions will be combined across all modules to search for global solutions that lie in the steady-state space for the fully combined network. Since PCA is a linear method, a steady-state solution space that is highly nonlinear may require more principal component vectors to accurately estimate the solutions. Nonlinear methods of dimensionality reduction, such as kernel PCA [32] or local linear embedding [33], may provide a more compact representation of steady-state solutions spaces in future iterations of the method.

The reduction procedure is illustrated with an example of a human platelet model comprising 4 interlinked signaling modules (Figure 2B). For each module, we used published reaction mechanisms and kinetic parameters to construct the module topologies [28]. Each topology was held fixed while the unknown CVs were sampled from empirically-defined distributions. For this step, we generated more than 10^9^ sets of initial guesses (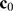) for each module, computed the initial value problem for each 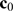 until a steady state was reached ( 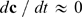 ), and selected only those steady-state CVs ( 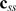 ) that were consistent with known concentrations. For example, the concentration of intracellular Ca^2+^ ([Ca^2+^]*_i_*, Figure 2B) in platelets is known to be ∼100 nM. Thus, only those 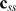 with [Ca^2+^]*_i_*≈100 nM were kept as part of the steady-state solution space for the Ca^2+^ balance module. This procedure was used to generate 10,000 steady state solutions for each module for subsequent reduction by PCA. A minimal set of principal component (PC) vectors (those capturing 90% or more of the variance in the solution set) were used as search directions in the final estimation step, in which the transient behavior of the perturbed steady-state was compared to experimental time-series data.

Interestingly, only a small fraction of initial guesses produce steady-state solutions that are also consistent with known concentration values. For example, it was previously shown that only 50,000 of 10^9^ initial guesses (0.005%) in the Ca^2+^ balance module (Figure 2B) met both requirements and were suitable for further analysis [28]. Among this set of CVs, marginal distributions for individual molecules were often confined to a narrow range of values. As an example, 80% of steady-state solutions for the calcium module contained <1000 IP_3_ molecules/cell, although initial guesses were sampled uniformly between 1 and 10^6^ molecules/cell. This observation shows that the kinetic topology of these molecular networks places very strong constraints on the range of concentrations that can exist at steady state. In biological terms, this suggests that fixed kinetic properties at the molecular level (e.g., IP_3_R and SERCA kinetics) can affect not only the dynamical features of a biochemical system but can also determine the abundance of chemical species and the compartmental structures that contain them.

### Merging Steady-State Modules

**Figure 3:**
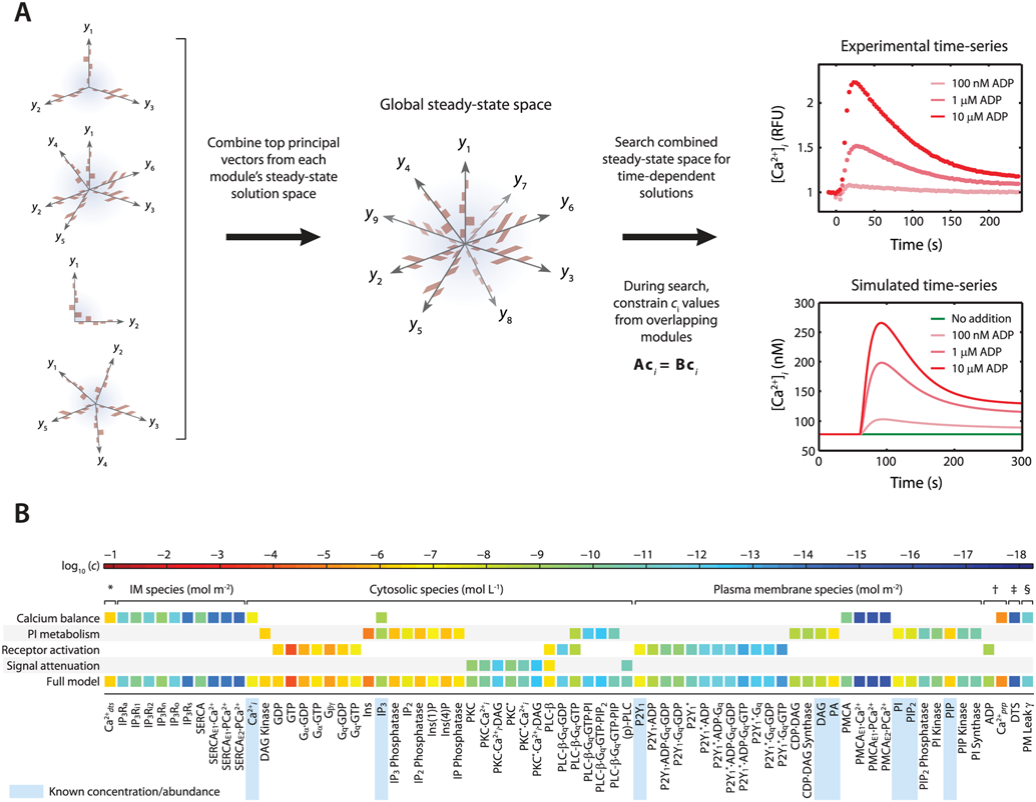
Assembly of full model from steady-state modules. (A) The full model is assembled by combining PCA-reduced, steady-state solution spaces from each module into a combined steady-state solution space. This global space is searched for full-length, steady-state solution vectors that satisfy both the steady-state requirements of each module and the desired time-dependent properties when the steady-state is perturbed (in this example, by increasing the concentration of the signaling molecule ADP and measuring the change in intracellular Ca^2+^ concentration). A simple linear constraint is imposed for every pair of modules that share a common molecule 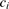 to ensure that steady-state solutions are consistent. (B) To assemble the platelet signaling model, a set of 16 PC vectors representing all 72 unknown variables in the model were used as search directions in a global optimization routine. The global solution space was searched for models with accurate dynamic behavior using experimental time-series data for ADP-stimulated Ca^2+^ release. Species are grouped according to compartment. Color values correspond to molar concentrations (mol/L or mol/m^2^) or as indicated: *DTS species (mol L^−1^). †Extracellular species (mol L^−1^). ‡DTS volume (L). §PM leak conductance/area (S m^−2^).

In the final step of the method, the full model is assembled by combining PCA-reduced, steady-state solution spaces from each module into a combined steady-state solution space for the entire system (Figure 3A). This global space is searched for full-length, steady-state solution vectors that satisfy both the individual steady-state requirements of each module and the desired time-dependent properties when the steady-state is perturbed (for example, by increasing the initial concentration of a signaling molecule). For the platelet signaling model, consisting of 77 reactions, 132 fixed kinetic parameters, and 70 species [28], a set of 16 PC vectors representing all 72 unknown variables (70 molecule concentrations, 1 compartment size, and 1 rate constant) in the model were used as search directions in a global optimization routine. The global solution space was searched for models with accurate dynamic behavior using experimental time-series data for ADP-stimulated Ca^2+^ release (Figure 3A). Equality constraints are imposed during optimization to maintain consistent concentrations of molecules that are present in more than one module. Specifically, for a steady-state space **A** represented by *m* PC vectors and a steady-state space **B** represented by *n* PC vectors, the projections of each space onto 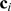 must be equal,
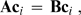
(4)where 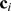 is the unit vector for the shared molecule, 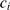. This condition forms a linearly-constrained optimization problem for which a number of efficient routines exist [22]. We used the Asynchronous Parallel Pattern Search (APPSPACK) to perform a derivative-free optimization of the platelet signaling model [24]. A least-squares objective function was used to score the difference between simulated (after perturbation of steady state) and experimental time-series data points. One of the high-scoring steady-state solution vectors for the full model is shown in Figure 3B, along with individual steady-state vectors for each of the four modules. This 72-dimensional vector (*i*) satisfies the homeostasis constraint in that it is a steady-state solution, (*ii*) is consistent with the known steady-state levels for 8 of the molecules in the 72-dimensional space, and (*iii*) predicts the entire dynamic Ca^2+^ and IP_3_ response of platelets exposed to ADP (0–100 µM). Additionally, rigid and flexible nodes (steady-state concentrations) in this 72-dimensional space were readily identified when a set of allowable steady-state solution vectors are compared [28].

### Applying the Method To Monitor Cellular Resting States

**Figure 4:**
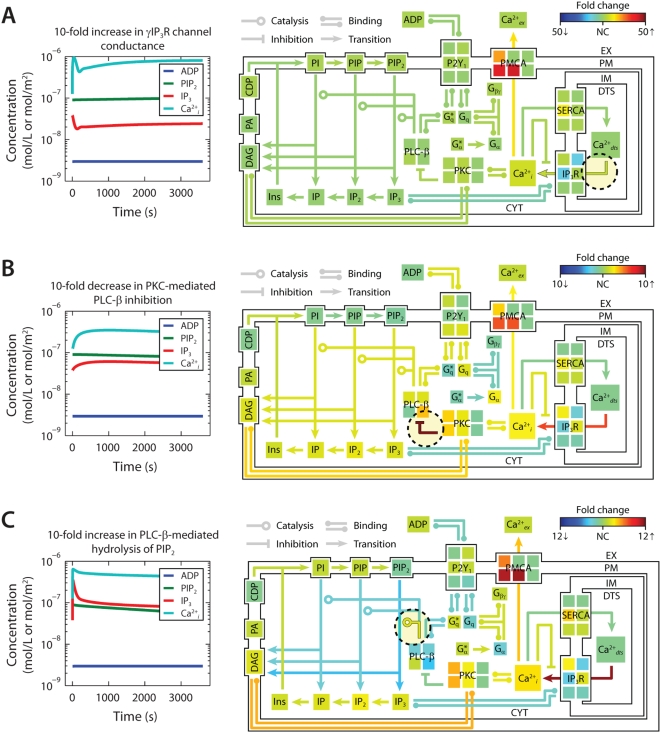
Shifts in steady-state profiles caused by kinetic perturbations. The steady-state platelet model was perturbed by changing selected kinetic parameters (±10-fold) and simulating for 1 h (*left panels*). After approaching a new steady state, the model concentrations and fluxes were determined relative to their original steady-state values and colored according to fold-change (*right panels*). Green indicates no change (NC) relative to initial flux/concentration. Red indicates a relative increase and blue indicates a relative decrease. Note that the color scale in each panel is normalized separately to maximize distinctions in fold change. New steady states were achieved after a (A) 10-fold increase in Ca^2+^ release through open IP_3_R channels ([28]), (B) 10-fold decrease in PKC-mediated inhibition of PLC-β, and (C) 10-fold increase in PIP_2_ hydrolysis (10-fold increase in *k*_cat_ of hydrolysis). Reactions with perturbed rate constants are circled and correspond to reaction mechanisms from [ref. 14]. (A) Ca^2+^ *_dts_* → Ca^2+^ *_i_*, (B) PKC*+PLC-β → PKC*+pPKC-β, (C) PLC-β*+PIP_2_ → PLC-β*+IP_3_+DAG.

Resting systems remain in a steady state by the coordinated action of opposing but balanced kinetic processes. Thus, in general, altering one ore more of these rate processes (e.g., increasing the catalytic rate of a reaction) should upset the balance of the system and cause it to adopt a new steady state. Various cell types have been shown to have altered steady-state properties because of mutations that affect the constitutive rates of reactions. For example, patients with type 1 diabetes harbor more Ca^2+^ ATPase activity in their platelets than healthy volunteers and experience high resting levels of intracellular Ca^2+^ [34]. In a separate case, a mutation within the tyrosine kinase domain of epidermal growth factor receptor causes significantly higher basal (growth factor-independent) tyrosine phosphorylation levels than the wild-type receptor [35]. Therefore, to examine the changes in steady-state properties caused by kinetic perturbations in our example model, we altered the rates of 3 important regulatory reactions and observed the system response to each perturbation. Each perturbation cause a brief adjustment phase lasting ∼200 s followed by a more gradual phase characterized by a new steady-state profile (Figure 4, *left*). After 1 hr of simulated time, steady-state concentrations and reaction fluxes were quantified relative to their original steady-state levels (Figure 4, *right*).

As expected, increasing the rate of Ca^2+^ release from intracellular stores resulted in higher cytosolic Ca^2+^ levels (7-fold increase) and 10-fold greater pumping activity by plasma membrane Ca^2+^ pumps (PMCA), although the new steady-state Ca^2+^ release flux remained relatively unchanged (Figure 4A). This perturbation also had little effect on the metabolism of phosphoinositides, as indicated by a predominantly green color. In a second perturbation, the inhibition of phospholipase C-β (PLC-β) activity by protein kinase C (PKC) was reduced 10-fold. Since PKC has a negative-feedback role in suppressing the platelet-stimulating activity of PLC-β, this perturbation caused a 2-fold increase in steady-state phosphatidylinositol 4,5-bisphosphate (PIP_2_) hydrolysis, elevated (inositol 1,4,5-trisphosphate) IP_3_ concentration, and accelerated Ca^2+^ release. Interestingly, the same reaction that was initially perturbed with a 10-fold decrease experienced a 10-fold increase in steady-state flux. This was a compensatory effect caused by the negative feedback loop involving Ca^2+^-regulated activity of PKC, a resulting new hypothesis that can be probed experimentally. In a third example, increasing the hydrolytic activity of PLC-β for the substrate PIP_2_ by 10-fold caused an expected stimulatory effect, raising intracellular calcium and steady-state levels of cytosolic inositol phosphates (IP_3_, IP_2_, and IP) between 2- and 3-fold. Interestingly, reaction fluxes for phosphoinositide hydrolysis were diminished, possibly due to substrate depletion. Taken together, these examples illustrate the system-wide effects of perturbations in the kinetic rate processes. The procedure could easily be extended to examine multiple simultaneous perturbations in both reaction rates and steady-state concentrations.

### Method Application, Computational Efficiency, and Extensions

We have presented a novel strategy for enumerating permissible steady-state solutions to fixed kinetic topologies and combining these solutions spaces to form large kinetic models. This is a practical strategy because kinetic parameters are commonly reported whereas absolute concentrations are not (see, for example, [6],[8],[9]). The method extends the capability to build “genome-scale” models [5],[10],[11],[36] to include nonlinear kinetic features. Through application of the method, we have also explored the implicit restrictions on steady-state solutions that can be imposed by the underlying kinetic structures within a system [4]. This is useful from a physiological standpoint since the regulation and distribution of molecular species in living systems is largely regulated by the coordinated action of synthetic, degrading, and transporting enzymes.

The proposed method requires the model to fulfill a steady-state assumption (i.e., the model must contain nontrivial steady states) even if the system is typically characterized by transient behavior. It is precisely this requirement that allows the model to have the dual functional behavior observed in many biological contexts, such as in cellular signaling responses. At very low levels of activating signal, the model remains at rest by quenching the low level of activating signal through feedback mechanisms or futile cycling. When activating signals are increased, the system responds with the appropriate transient signaling behavior. As an example, a human platelet must remain quiescent under normal circulating conditions, tolerating a number of fluctuations in its surrounding chemical and physical environment. In the presence of the appropriate stimulus, however, it must be able to respond rapidly to bleeding conditions and trigger a precise program of molecular signaling events. Developing a mathematical model that is consistent with two or more biological behaviors is analogous to writing a set of equations that has multiple solutions, each dependent on a given set of initial conditions and parameter values.

Our approach differs critically from metabolic flux analysis and previous genome-scale metabolic network reconstructions [5],[16] because it accommodates nonlinear terms that describe the dynamic behavior of each reaction in the system. Previous large-scale network reconstructions typically use a stoichiometry matrix to represent the gross flux of metabolites in the system [17]. Here, we have preserved the mathematical form of each kinetic rate equations as reported in the literature, allowing models to be built from existing data in a “bottom-up” fashion [10] while still allowing calibration to whole-system experimental data. This feature will substantially improve the accuracy of dynamical system simulation and parameter estimation.

Additional computational savings are provided through modularization. When estimating modules of modest size (5 or less unknown concentrations), we use a brute-force Monte Carlo approach to densely sample the feasible space of initial conditions. Larger networks (20 or more unknowns) cannot be efficiently searched in this brute-force manner, but can be built piecewise by combining subspaces of smaller size that have been densely sampled. Using the naïve Monte Carlo approach, estimating *n* free parameters is exponential in *n*. By dividing these parameters into *k* independent networks, each with *n*/*k* free parameters, the estimation procedure becomes exponential in *n*/*k* and thus more tractable. By assembling the entire system from smaller, more manageable kinetic modules, data may be used to test the functionality of individual modules before incorporating them into the entire system. In several cases, this approach was shown to offer a substantial computational benefit (e.g., reducing the global search space by over 10,000-fold) by simply requiring a steady-state solution with known subcomponent values. The search space can be reduced further by principal component analysis if there is correlation between free parameters within a module. This was found to be the case for enzymes that have opposing regulatory roles; increasing the levels in one enzyme required a similar increase in the other in order to preserve homeostasis. Lastly, modules sharing common components must hold the same value for that component, which imposes an additional constraint on the steady-state solutions (equation (4)).

As presented, the method exploits known kinetic parameters to restrict unknown concentrations due to kinetic interactions. However, the method is equally valid for estimating unknown kinetic parameters and/or utilizing known concentrations. Both concentrations and kinetic parameters appear indistinguishably as nonlinear terms in the ordinary differential equations that describe the system (Figure 1B). Hence, it does not matter which types of values are known and which are estimated; the procedure is valid for mixed or incomplete sets of unknown values. The use of qualitative data may also be exploited by the method. For example, beginning with a large set of steady-state solutions for a given module, the size of the set may be reduced by determining which solutions in the set contain some qualitative behavior or function. In a previous application of the method [17], a set of 10^9^ steady-state solutions representing calcium balance in a resting platelet were divided into 3 groups, according to their qualitative response to increased IP_3_ concentration (low, mild, and high response). Using this technique, the functional testing of steady-state modules may be used to eliminate a large subset of the original steady-state solution set. As another example, one may use data from a Western blot to establish the relative abundance between two proteins in the model. This qualitative information may be used to filter the steady-state solutions to a reduced set that is consistent with experimental results. This kinetically-driven, constraint-based approach, which combines a homeostasis requirement with known kinetic parameters and cellular concentrations, naturally enforces numerical limits on unknown system quantities.
